# Insights on Polyidide Shuttling of Zn-I_2_ Batteries by I_3_^−^/I^−^ Electrolytes Based on the Dual-Ion Battery System

**DOI:** 10.3390/nano15100738

**Published:** 2025-05-14

**Authors:** Xingqi Chang, Andreu Cabot

**Affiliations:** 1 Catalonia Institute for Energy Research-IREC, Sant Adrià de Besòs, 08930 Barcelona, Spain; xchan@irec.cat; 2 Facultat de Química, Universitat de Barcelona, Carrer de Martí i Franquès, 08028 Barcelona, Spain; 3 ICREA Pg. Lluis Companys, 08010 Barcelona, Spain

**Keywords:** Zn-I_2_ battery, polyiodide shuttling, dual-ion battery, I_3_^−^/I^−^ conversion

## Abstract

The rechargeable zinc-iodine (Zn-I_2_) battery is a promising energy storage system due to its high theoretical capacity, low cost, and safety. So far, most researchers agree that the poor electrical conductivity of iodine and the shuttling of polyiodide lead to a rapid decrease in capacity and low coulombic efficiency (CE) during cycling, which seriously hinders their further development and application. Herein, to understand the polyidide shuttling effects in Zn-I_2_ battery, we utilize I_3_^−^/I^−^ electrolytes as the active capacity source coupled with carbon cloth, devoid-of-iodine (I_2_) loading cathode, to simulate the behavior of the shuttling of polyidide in the Zn-I_2_ battery, based on the concept of a dual-ion battery system. Experiments show that these batteries exhibit a specific capacity of 0.24 mAh·cm^−2^ at 1.0 A·cm^−2^ and 0.2 mAh·cm^−2^ at 20 A·cm^−2^, corresponding to 1.0~1.3 mg active mass of I_2_, based on the 2I^−^/I_2_ redox couple (221 mAh·g^−1^). It is noteworthy that the inclusion of polyiodide enhances the electrochemical and redox activity, which is advantageous for electrochemical performance; however, it is limited to the polyiodine reduction on the Zn surface (Zn + I_3_^−^ → 3I^−^ + Zn^2+^).

## 1. Introduction

Rechargeable aqueous metal-ion batteries with neutral to mildly acidic electrolytes hold great promise for large-scale energy storage due to their intrinsic safety, long cycle life, low cost, and environmentally sustainable design [1,2], such as zinc-iodine (Zn-I_2_), zinc-manganese dioxide (Zn-MnO_2_), and zinc-vanadium pentoxide (Zn-V_2_O_5_)[3,4]. Among them, zinc-iodine (Zn-I_2_) batteries stand out for their economic viability, as zinc is abundant in the Earth’s crust and iodine is plentiful in ocean reserves [5,6].

Their appeal is further enhanced by the high theoretical capacities of zinc (820 mAh·g⁻^1^) and iodine (211 mAh·g⁻^1^) [7,8] and by the full electron transfer during redox reactions, which contributes to a flat voltage plateau, an advantage over conventional intercalation-type cathodes [9]. However, the practical application of Zn-I_2_ batteries is hindered by their limited cycle life and the shuttling of polyiodide species [7].

In aqueous electrolytes, Zn-I_2_ batteries operate through a reversible redox reaction between iodine (I_2_) and iodide (I^−^), forming intermediate polyiodides, such as I_3_^−^ and I_5_^−^ (I^−^ + I_2_ ⇌ I_3_^−^; I_3_^−^+ I_2_ ⇌ I_5_^−^) [7,10]. The uncontrolled diffusion of these intermediates leads to several detrimental effects. The conversion between I^−^ and I_3_^−^ at the cathode is kinetically sluggish due to a high energy barrier [11,12]. Moreover, the rapid diffusion of I_3_^−^ into the electrolyte causes it to shuttle to the zinc anode under the potential, where it undergoes reduction (Zn + I_3_^−^ → Zn^2+^ + I^−^), depleting active iodine species and resulting in self-discharge and capacity fading [13,14].

To mitigate the shuttle effect, porous inert materials such as activated carbon, graphene, and MXene have been explored for polyiodide confinement, leveraging their high surface areas for enhanced adsorption [15,16]. However, the relatively weak interactions between polyiodides and these materials are insufficient to fully suppress shuttling during extended cycling, leading to persistent self-discharge and limited progress toward shuttle-free Zn-I_2_ batteries [17,18]. Additionally, the disordered pore structures and electrochemical inertness of many carbon-based hosts limit iodine utilization and ion transport, while also hindering efficient I_2_/I^−^ redox conversion [10,19]. These issues result in sluggish reaction kinetics and reduced rate performance and cycling stability [20].

Revisiting the subject, Zn-I_2_ battery systems often incorporate iodine-based additives, such as potassium iodide (KI), sodium iodide (NaI), zinc iodide (ZnI_2_), and lithium iodide (LiI), to enhance electrochemical performance [9,21]. These additives improve both capacity and cycling stability. Their inclusion, combined with the redox activity of iodine species and Zn^2+^ deposition/dissolution processes, gives rise to a class of dual-ion battery (DIB).

DIBs, which have attracted growing attention in recent years, are characterized by the active participation of both cations and anions from the electrolyte in the redox processes on the cathode and anode sides. While the mechanism of charge separation during diffusion is analogous to the operational mechanism of supercapacitors, where physical charge storage processes occur, the storage and release of ions in DIBs are based upon Faradaic charge storage reactions, such as intercalation or insertion into a host material, as well as alloying or deposition reactions. These latter processes can occur on the anode side for cation storage. This distinguishes them from traditional “rocking chair” batteries, since DIB ions are stored within the electrolyte phase when the cell is in a discharged state [22]. In contrast, the active species in rocking chair cells are initially stored in a host material (for example, in the cathode of a commercial Li-ion battery during the discharge state), and, aside from interphase formation, the electrolyte primarily functions as a charge carrier between the anode and cathode [23].

A representative example is the Al-graphite DIB (AGDIB) developed by Tang’s group, using the 4.0 M LiPF_6_ in carbonate electrolyte [24]. In this system, hexafluorophosphate (PF_6_^−^) ions undergo intercalation/deintercalation into the graphite cathode, while an alloying reaction occurs between aluminum and lithium at the anode. The term “dual-ion” reflects the cooperative function of both anions and cations as charge carriers. In the context of Zn-I2 aqueous batteries, it is essential not only to mitigate the polyiodide shuttle effect but also to investigate its underlying mechanisms to better understand polyiodide transport behaviors [25]. Moreover, there is a lack of definitive clarification regarding the extent to which the introduced iodine ion additives enhance the capacity and their corresponding influence on the overall Zn-I_2_ battery. Furthermore, because of the presence of water-soluble zinc iodide, the production of polyiodide during the battery cycle is unavoidable. Consequently, it is imperative to assess the role of iodide in Zn-I_2_ batteries.

To this end, we assembled a dual-ion zinc-iodine battery (DIZIB) using a mixed electrolyte containing 0.1 M ZnI_2_ and 0.1 M I_2_ in 2.0 M ZnSO_4_. This configuration emulates the shuttling of I^−^ and I_3_^−^ species, with Zn foil as the anode and carbon cloth (Φ = 12 mm) as the cathode.

The resulting DIZIB exhibits excellent rate capability, delivering specific capacities of 0.24 and 0.20 mAh·cm^−2^, equivalent to 1.2 mg and 1.0 mg of I_2_, respectively, at high current densities of 10 and 20 A·cm^−2^. These capacities are based on the theoretical value of 211 mAh·g^−1^ for the I^−^/I_2_ redox couple. The elevated iodine concentration in the electrolyte not only enhances capacity but also lowers charge-transfer resistance and improves ion diffusion within the cathode. This highlights the need for a more comprehensive understanding and rationalization of polyiodide shuttling and its impact on capacity contribution in Zn-I_2_ battery systems.

## 2. Experimental Section

### 2.1. Chemical

Whatman glass fiber (Whatman, GF/A, Munich, Germany), zinc sulfate heptahydrate (ZnSO_4_·7H_2_O, 99%), and zinc iodide (ZnI_2_, 99%) were purchased from Sigma-Aldrich (Schnelldorf, Germany). Mili-Q water (18 MΩ cm^−^) was supplied by a Purelab Flex from Elga (High Wycombe, Buckinghamshire, United Kingdom). Ethanol was of analytical grade and obtained from Lestlab Delivering Solutions (Sant Cugat del Vallès, Spain). Zinc foil (1 mm of thickness, 99%) and carbon cloth were purchased from Guangdong Canrd Co., Ltd. (Dongguan, China). All the chemicals were used as received, without further purification.

### 2.2. Electrochemical Measurements

Electrochemical tests of the batteries were performed on CR2032-type coin cells. The cell was composed of zinc foil and carbon cloth, which was punched into 12 mm circular foil as an anode electrode, and the glass fiber with a diameter of 16 mm was used as a separator. Given that the battery capacity is contingent upon the electrolyte and that there exists no active material within the cathode electrode, the area capacity is employed as a standard by incorporating an equivalent volume of electrolyte. Aqueous solutions containing 2.0 M ZnSO_4_ + 0.1 M ZnI_2_ and 2.0 M ZnSO_4_ + 0.1 M ZnI_2_ + I_2_ were used as electrolytes (80 μL), marked as EL and EL-I_2_, respectively. Five batteries were utilized to conduct parallel experiments for similar tests. The variation in the capacities of the five batteries was maintained within five percent, with the median value employed as the target data. The coin cells were assembled in air and measured at room temperature. The symmetrical battery was assembled using carbon cloth as both electrodes, using the same electrolytes. Before cycling, all coin cells were aged for 1 h to ensure sufficient electrolyte penetration. Galvanostatic charge/discharge (GCD) tests were carried out on the Neware BES-4008 battery test system with a potential range set between 0.6 and 1.6 V (vs. Zn^2+^/Zn). Cyclic voltammetry (CV) tests were performed on a BCS-810 battery tester from Bio-Logic at different scan rates with a potential range set from 0.8 to 1.6 V (vs. Zn^2+^/Zn). Electrochemical impedance spectroscopy (EIS) measurements were carried out using a sinusoidal voltage with an amplitude of 10 mV and a frequency from 10 kHz to 0.1 Hz.

## 3. Results and Discussion

### 3.1. Dual-Ion Zn-I_2_ Battery

**Figure 1:**
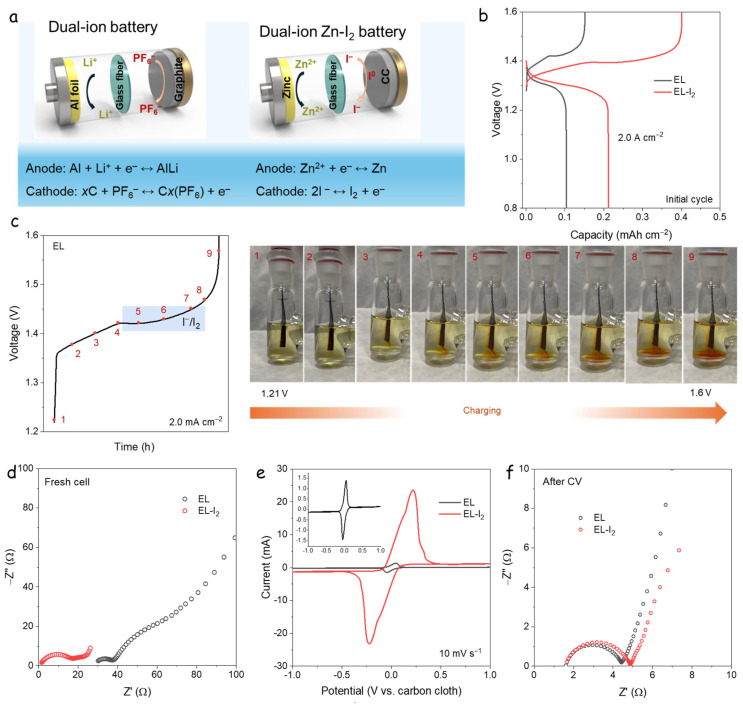
(**a**) Schematic diagram of AGDIB and DIZIB; (**b**) GCD curves of the initial cycle at 2.0 Acm^−2^; (**c**) Charge curve and corresponding color change of the carbon cloth cathode side at different times; (**d**–**f**) Asymmetric carbon cloth/carbon cloth battery system tests: (**d**) EIS spectra of fresh cells; (**e**) CV curves at 10 mV·s^−1^; and (**f**) EIS spectra after CV tests.

Figure 1a presents a schematic comparison of the AGDIB and the DIZIB [18]. In the DIZIB, carbon cloth (Φ = 12 mm) serves as an iodine-free cathode, paired with a zinc foil anode. In this study, the widely utilized zinc sulfate electrolyte serves as the foundation for the source of active capacity through the incorporation of various iodine species substrates [21]. The baseline electrolyte, comprising 2.0 M ZnSO_4_ and 0.1 M ZnI_2_ (denoted as EL), is compared with a modified electrolyte containing an additional 0.1 M I_2_ (denoted as EL-I_2_). This composition is designed to promote the formation of I₃⁻ species via the equilibrium reaction I^−^ + I_2_ ⇌ I_2_⁻. The progressively darker brown color of the EL-I_2_ solution with increasing iodine concentration (Figure S1) visually confirms the increased presence of I_3_^−^.

Figure 1b shows the GCD curves of the DIZIBs at 2.0 A·cm⁻^2^ during the initial cycle. The EL-based system delivers a specific capacity of 0.11 mAh·cm⁻^2^ with a coulombic efficiency (CE) of 82%. In contrast, the EL-I_2_ system achieves a higher capacity of 0.217 mAh·cm⁻^2^, albeit with a lower CE of 51%, indicating the successful operation of a DIB mechanism via I^−^/I_3_^−^ redox activity. During discharge, both systems display a voltage plateau at ~1.25 V vs. Zn^2+^/Zn, corresponding to the I^−^/I_2_ redox couple characteristic of Zn-I_2_ batteries. On charging, the EL-I_2_ system exhibits additional irreversible plateaus at ~1.3 and ~1.4 V, attributed to the oxidation and conversion states of I_3_^−^ species.

To provide visual confirmation of the I^−^/I_3_^−^ redox processes, Figure 1c presents an in situ colorimetric analysis (photograph in Figure S2), using the 2.0 M ZnSO_4_ + 0.1 M ZnI_2_ electrolyte. Iodide ions are colorless in solution, while I_2_ and I_3_^−^ species exhibit a distinct brown coloration. During charging, brown flocs form near the carbon cloth electrode as voltage increases, as seen in Figure 1c (points 4–9), confirming the generation of I_2_/I_3_^−^. These observations demonstrate that the redox reactions involving I^−^/I_2_ and I^−^/I_3_^−^ occur at the cathode, along with brown floccules spread, while Zn^2+^ deposition proceeds at the anode, consistent with a DIB mechanism [26].

To further investigate the redox activity, symmetric cells using carbon cloth (Φ = 12 mm) as both electrodes were assembled in a coin cell. EIS of fresh cells (Equivalent electrical circuits are shown in Figure S3) revealed that EL (2.0 M ZnSO_4_ + 0.1 M ZnI_2_) exhibits higher intrinsic resistance (as seen from the high-frequency x-intercept), whereas EL-I_2_ (2.0 M ZnSO_4_ + 0.1 M ZnI_2_ + 0.1 M I_2_) shows significantly lower resistance, indicating enhanced electrochemical activity. Due to the symmetric cell system and the reactions of I^−^/I_3_^−^ occurring at the electrolyte, it is difficult to fit and accurately measure the resistance of different parts. Therefore, this test mainly focuses on the overall resistance of the battery. The cell resistance is represented at high frequency in EIS results. Consequently, polyiodine reduced the cell resistance. Figure 1d shows CV curves at a scan rate of 10 mV·s⁻^1^. The EL-I_2_ system exhibits a response current nearly 20 times greater than that of EL, confirming the high redox activity of I_3_^−^ on carbon electrodes. After CV cycling, EIS measurements (Figure 1f) show reduced impedance for both electrolytes compared to the fresh state, displaying similar semicircles and intrinsic resistance in both cells, likely due to the establishment of a redox equilibrium between I_3_^−^ and I^−^.

Overall, the AGDIB represents a quintessential dual-ion battery system, utilizing non-aqueous LiPF_6_ within the carbonate electrode. This system functions through the simultaneous reaction of cations (Li^+^) and anions (PF_6_^−^) entering the anode and cathode, respectively, during the charging process. Subsequently, in the discharge process, Li^+^ and PF_6_^−^ ions return from the electrode materials to the bulk electrolyte concurrently. In the charging process of the DIZIB, the reduction of I^−^/I_3_^−^ anions to I_2_ occurs, alongside the deposition of Zn^2+^ onto the surface of the Zn foil. Conversely, during the discharge process, I_2_ is oxidized to I^−^ anions, while the dissolution of Zn^2+^ diffuses back into the electrolyte.

### 3.2. Electrochemical Performance of DIZIBs

**Figure 2:**
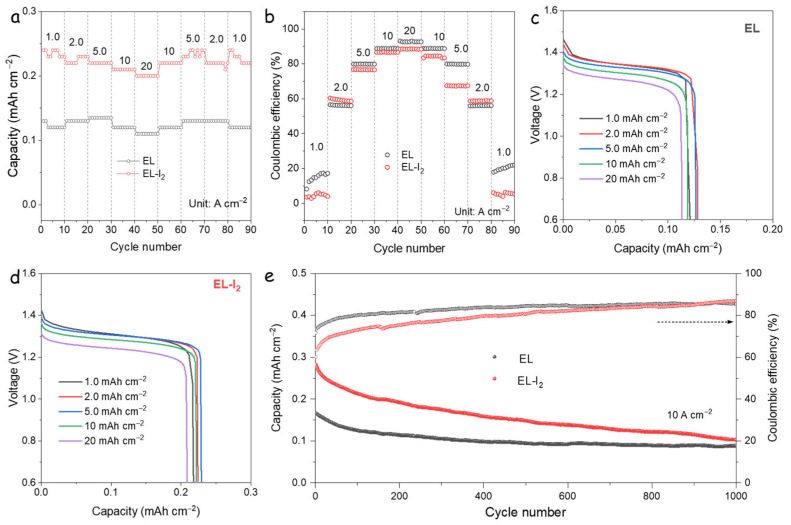
(**a**) Rate performance and (**b**) corresponding CE of EL and EL-I_2_ cells; (**c**) GCD curves of EL and (**d**) EL-I_2_ cells; (**e**) Cycling stability tests at 10 A·cm^−2^.

Further insights into the electrochemical performance were obtained through rate capability tests at varying current densities (Figure 2a). The results show that the EL-I_2_ cell delivers nearly twice the specific capacity of the EL cell across all tested rates. Figure 2b displays the corresponding CEs. As the current increases, both cells exhibit improved CE, indicating that side reactions involving iodide and polyiodide species are more prominent at lower currents. Notably, although EL-I_2_ achieves higher capacity, its CE is consistently lower than that of EL. This suggests that the introduction of I_3_^−^ or excess I_2_ does not enhance CE and may, in fact, intensify parasitic reactions, likely due to the elevated concentration of iodine-containing species. According to the reversible reaction I_2_ + I^−^ ⇌ I_3_^−^, high-rate cycling favors the rapid reduction of I^−^ to I_2_ and promotes the dissociation of polyiodide species, which mitigates their accumulation. As a result, the CE values of EL and EL-I_2_ converge at higher current densities, where the influence of polyiodide shuttling is diminished.

Figure 2c–d show the discharge voltage profiles of EL and EL-I_2_ at various current densities. Both cells exhibit a consistent voltage plateau, corresponding to the I^−^/I_2_ redox couple [27]. While the overall voltage behavior is similar, EL-I_2_ consistently demonstrates higher capacity. For instance, at 20 A·cm⁻^2^, EL-I_2_ delivers 0.21 mAh·cm⁻^2^, compared to 0.11 mAh·cm⁻^2^ for EL. Based on the theoretical capacity of I_2_ (211 mAh·g⁻^1^), this corresponds to I_2_ loadings of approximately 1.13 mg and 0.56 mg for EL-I_2_ and EL, respectively. The long-term cycling performance at 10 A·cm⁻^2^ is depicted in Figure 2e. Both cells exhibit rapid capacity fading, likely due to low CE, which accelerates the loss of active material.

### 3.3. Kinetic Analysis

**Figure 3:**
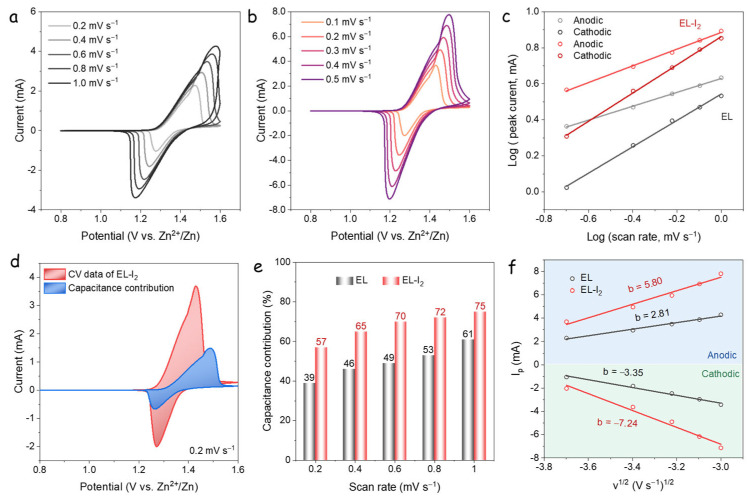
(**a**,**b**) CV curves of (**a**) EL and (**b**) EL-I_2_ at various scan rates. (**c**) Linear relationship between log (*I_p_*) vs. log (*v*). (**d**) CV curves of the EL-I_2_ cell with the capacitance contribution at a scan rate of 0.2 mV s^−1^. (**e**) Ratios of capacitance at various scan rates. (**f**) The relationship between the peak current (*I_p_*) and the square root of scan rate (*v*^1/2^) of anodic and cathodic peaks.

To further elucidate the differences in rate capability between the two cells, the apparent diffusion coefficient of Zn^2+^ and I^−^/I_3_^−^ ions, as well as the contribution from capacitive processes, were evaluated based on CV measurements. Figure 3a,b show the CV curves of EL and EL-I_2_ cells at scan rates from 0.2 to 1.0 mV·s^−1^. Both systems exhibit well-defined cathodic and anodic peaks, indicative of reversible redox behavior. To qualitatively assess the capacitive contribution, log-log plots of peak current versus scan rate were constructed, based on Equation (1):

(1) $$i_{p}=av^{b}$$

According to Equation [1], the current response increases with the sweep speed due to the capacitive contribution. Meanwhile, the polarization enlarges as the sweep speed increases, alongside the redox peak potential difference [28]. This analysis allows the differentiation between diffusion-controlled and surface-capacitive processes: a slope near 0.5 corresponds to a diffusion-controlled process, while a slope approaching 1.0 indicates a capacitive-dominated response [29]. The *b*-values, derived from the slope of the log(*i*) versus log(*v*) plots, were calculated to be 0.39 and 0.46 for the anodic peaks, and 0.73 and 0.78 for the cathodic peaks of the EL and EL-I_2_ cells, respectively (Figure 3c) [30,31]. These results suggest that both systems exhibit similar electrochemical behavior during the redox processes. The anodic *b*-values, being closer to 0.5, indicate that the oxidation process is predominantly diffusion-controlled. In contrast, the cathodic *b*-values, approaching 1.0, suggest a more capacitive contribution during reduction [32]. Overall, the EL and EL-I_2_ systems demonstrate comparable trends in ion storage and transport dynamics across the redox processes.

Furthermore, the fractional contribution of current at a given potential can be quantitatively determined using Equation (2):

(2) $$i_{p}(V)={\;k}_{1}v+{{\;k}_{2}v}^{1/2}$$

where the *k*_1_*v* represents the surface-controlled (capacitive) contribution and and *k*_2_*v*^1/2^ corresponds to the diffusion-controlled process [32]. Using this method, the capacitive contribution for the EL-I_2_ cell at a scan rate of 0.2 mV·s⁻^1^ was calculated to be 57% of the total current response (Figure 3d).

The evolution of the capacitive contribution with scan rate is summarized in Figure 3e. For the EL cell, the capacitive contributions were 39%, 46%, 49%, 53%, and 61% at scan rates of 0.2, 0.4, 0.6, 0.8, and 1.0 mV·s⁻^1^, respectively. In comparison, the EL-I_2_ cell exhibited higher capacitive contributions of 57%, 65%, 70%, 72%, and 75% at the corresponding scan rates. These results highlight the significant surface-controlled behavior in the EL-I_2_ system, which can be attributed to the presence of I_2_ and polyiodides enhancing pseudocapacitive interactions. The use of an I_2_-free carbon cloth cathode ensures that these capacitive enhancements originate from the electrolyte, underscoring the role of iodide/polyiodide redox activity in achieving high capacity and fast charge-discharge performance.

In Figure 3f, the apparent diffusion coefficient of ions was estimated from the relationship between the anodic/cathodic peak current (*I_p_*) and the square root of scan rate (*V*^1/2^) [33,34]:

(3) $$i_{p}=2.69\times 10^{5}An^{3/2}C_{0}D_{ion}^{1/2}n^{3/2}v^{1/2}$$

where *n* denotes the number of electrons in the redox reaction per molecule, *A* represents the geometric electrode surface area, and *C*_0_ indicates the molar concentration of ions in both the cathode and anode. In DIZIBs with multiple ions, the diffusion coefficient’s magnitude can be approximated by the slope’s absolute value. The EL cell shows slopes of 2.81 and −3.35, while EL-I_2_ cell presents values of 5.80 and −7.24 for anodic and cathodic peaks, respectively. Thus, EL-I_2_ shows higher ion diffusion coefficient, indicating that I_3_^−^ anions enhance diffusion in Zn-I_2_ batteries, consistent with the EIS results.

### 3.4. Electrochemical Behavior of High Concentration I-Additives

**Figure 4:**
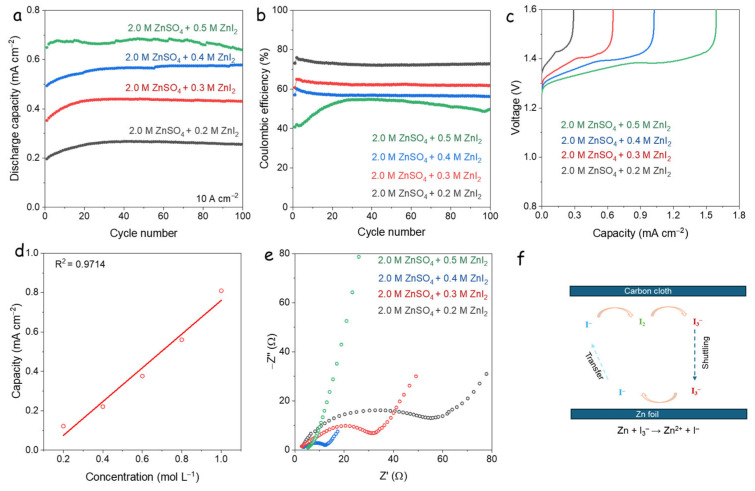
(**a**) Cycling performance of the different concentrations of additives (e.g., 0.2, 0.3, 0.4, 0.5 M) at 10 A·cm^−2^, (**b**) corresponding CE, and (**c**) charge curves; (**d**) linear relationship between iodide ion concentration and discharge capacity; (**e**) EIS spectra; (**f**) Schematic diagram of self-discharge and low CE.

To investigate the influence of iodide concentration on the electrochemical performance of DIZIBs, varying concentrations of ZnI_2_ additives (0.2, 0.3, 0.4, and 0.5 M) were tested. The cycling performance at a high current density of 10 A·cm⁻^2^ is shown in Figure 4a, while the corresponding CEs are presented in Figure 4c. Due to the corrosive nature of iodide ions on the Zn anode [35], the initial 100 cycles were consistently utilized as the standard for comparing capacities in various ZnI_2_ concentration cells. The results indicate that increasing ZnI_2_ concentration leads to higher discharge capacities but concurrently results in reduced CE. This trade-off suggests that while additional I^−^ ions enhance capacity through increased redox activity, they also promote parasitic reactions that degrade efficiency.

The charge profiles at the 5th cycle (Figure 4b) display two distinct voltage plateaus at approximately 1.35 V and 1.40 V. Based on previous reports, the 1.35 V plateau corresponds to the I^−^/I_2_ redox couple, while the 1.40 V plateau is attributed to side reactions involving I_3_^−^ or overoxidized iodine species. The lower CE is, thus, likely associated with these irreversible processes at higher potentials.

Further analysis revealed a strong linear correlation between iodine concentration (0.2 to 1.0 M) and discharge capacity, as shown in Figure S4, with a high correlation coefficient (R² = 0.9714; Figure 4d). This linearity confirms that the discharge capacity of DIZIBs is directly dependent on the concentration of electroactive iodine species in the electrolyte. EIS results (Figure 4e) further support this trend, showing that higher iodine concentrations significantly reduce electrolyte resistance. This is attributed to the increased availability of redox-active I^−^ ions, which enhance ionic conductivity and facilitate faster charge transfer processes.

### 3.5. Self-Discharge Analysis

The reduction of iodide ions at the zinc foil surface, described by the reaction Zn + I_3_^−^ → Zn^2+^ + I^−^, raises important considerations regarding the stability and persistence of polyiodide species in the electrolyte, particularly within the confined environment of a coin cell, where the diffusion distance is minimized. As shown in Figure S5, the electrolyte in contact with a zinc foil became completely colorless after standing overnight, indicating the full reduction of polyiodide species. This observation confirms that polyiodides are not stable in the presence of zinc and are rapidly consumed via spontaneous redox reactions, leading to the loss of active iodine species and contributing to self-discharge. The standard potential equations are shown below:

Zn → Zn^2+^ + 2*e*^−^ E° = −0.76 V vs. SHE (4)

I_3_^−1^ + 2*e*^−^ → 3I^−^ E° = 0.52 vs. SHE (5)

Zn + I_3_^−1^ → Zn^2+^ + 3I^−^ E*° =* 1.30 V vs. SHE (6)

These standard potentials suggest that these reactions are spontaneous. Due to the short diffusion distance of the coin cell, the polyiodide ions can easily shuttle to the Zn side and be reduced, as schematically shown in Figure 4f. Therefore, the plateau at 1.40 V is related to the I_3_^−^/I^−^ redox couple at the anode side, which causes the low CE.

Self-discharge occurs not only as a result of thermodynamic stability but also due to the double-layer Gouy-Chapman model. In a rocking chair Zn-I_2_ battery, zinc ions depart from the cathode electrode material and migrate to the anode electrode surface during the charging process. Concurrently, an increased formation of polyiodides may occur on the cathode electrode surface. A portion of these polyiodides enters the electrolyte, establishing a Gouy-Chapman model [36], and distances itself from the cathode electrode through various ion diffusion interactions. Given the short distance between the anode and cathode electrodes in a coin cell, diffusion to the Zn anode side is facilitated, resulting in self-discharge.

Furthermore, the porosity of the separator has minimal impact, as the distance between the positive and negative electrodes is considerably short. Consequently, determining an effective method to match the anion in order to mitigate the diffusion layer of polysulfide emerges as a highly effective strategy, based on the Gouy-Chapman model.

### 3.6. Prospects of Strategies for Mitigating Self-Discharge in Zn-I_2_ Batteries

In the context of self-discharge in zinc-iodide batteries, it is essential to consider the liquid-flow Zn-I_2_ battery system characterized by stable electrochemical performance [37]. This flow Zn-I_2_ system’s internal structure incorporates two electrolytes, with the most significant feature being the employment of cation-selective permeable membranes that mitigate the shuttling of various iodides. Consequently, the most effective strategy involves the utilization of an anion-suppressing separator or modification of the separator. In addition, an electrolyte strategy can be employed to impede the migration of polyiodides, based on the Gouy-Chapman model. Lastly, the formation of a layer on the surface of the zinc foil, analogous to that used in lithium-ion batteries, creates a solid electrolyte interphase (SEI) that provides an insulating effect [38], thereby preventing polysulfide from reaching the surface of the zinc foil.

In the design of cathode materials, sulfur carriers can be developed utilizing nanostructured configurations, including hollow spheres, metal-organic frameworks (MOF), and covalent organic frameworks (COF), among other structures. Additionally, the pursuit of novel adsorption mechanisms and the enhancement of the catalytic efficacy of iodine carriers represents a commendable alternative, drawing inspiration from lithium-sulfur batteries [39].

### 3.7. Distinction Between Dual-Ion and Rocking Chair Zn-I_2_ Batteries

In a pure rocking chair Zn-I_2_ battery, assuming no polyiodide ions are generated, the anode consists of Zn, and the cathode consists of iodine-containing material. During the discharge process, the Zn^2+^ ions dissolve from the Zn anode and transfer into the cathode side, reacting with the iodine. For the charge process, the Zn^2+^ is released from the ZnI_2_ and deposited on the Zn anode. The charge process could be concluded as follows:

Anode: Zn^2+^ + 2*e*^−^ = Zn (7)

Cathode: ZnI_2_ = I_2_ + 2*e*^−^ + Zn^2+^ (8)

Overall cell reaction: ZnI_2_ = Zn + I_2_ (9)

In contrast, for the DIZIB, the cathode is iodine-free carbon cloth, while the I^−^/I_3_^−^ anion and cations are stored in the electrolytes. During the charge process, the I^−^/I_3_^−^ are reduced to I_2_ on the cathode sides, and the Zn^2+^ deposition occurs on the Zn anode, simultaneously. Meanwhile, for the discharge process, the I_2_ is oxidized to I^−^ anions, and the dissolution of Zn^2+^ diffuses back to the electrolyte, respectively. The charge process is shown in the following:

Anode: Zn^2+^ + 2*e*^−^ = Zn^2+^ (10)

Cathode: 2I^−^ = I_2_ + 2*e*^−^ (11)

Overall cell reaction: Zn + 2I^−^ = Zn^2+^ + I_2_ (12)

## 4. Conclusions

In summary, we introduce a DIB concept designed to simulate the polyiodide shuttling phenomenon by utilizing iodide-based electrolytes and an iodine-free cathode. The resulting DIZIB, based on I^−^/I_3_^−^ redox chemistry, demonstrates a high areal discharge capacity exceeding 0.2 mAh·cm⁻^2^ at a current density of 10 A·cm⁻^2^ (Φ = 12 mm), with a CE of approximately 80%, corresponding to the utilization of ~1.2 mg of I_2_. Furthermore, this phenomenon may clarify the reasons why specific Zn-I_2_ batteries are documented to demonstrate performance that exceeds the theoretical capacity, which can be ascribed to the integration of dual-ion batteries within the system. Concurrently, the presence of polyiodide does not impact the discharge capacity of the battery. Consequently, the zinc-iodine battery sustains a comparatively stable capacity retention rate. Notably, the inclusion of I_3_^−^ as an additive significantly reduces the internal resistance and enhances the redox activity of the 2I^−^/I_2_ couple, contributing positively to electrochemical performance. However, due to the spontaneous reduction of polyiodides at the Zn anode (Zn + I_3_^−^ → Zn^2+^ + I^−^), the CE remains limited, highlighting the need to address this issue for practical applications. Given the widespread use of iodide/polyiodide-based additives in Zn-I_2_ battery research, we advocate for the strategic implementation of high-loading I_2_ cathodes to maximize capacity while minimizing parasitic reactions. Additionally, low-current testing proves to be an effective diagnostic tool for qualitative polyiodide shuttling and to distinguish true capacity from contributions due to soluble I^−^/I_3_^−^ species. Looking ahead, future research should focus on electrolyte engineering to suppress polyiodide reduction at the zinc anode, particularly in systems employing iodide/polyiodide additives. Such strategies are essential for advancing the performance and reliability of Zn-I_2_ batteries [18].

## Data Availability

The original contributions presented in this study are included in the article. Further inquiries can be directed to the corresponding author.

## Supplementary Materials

https://www.mdpi.com/article/10.3390/nano15100738/s1, Figure S1. Photograph of vials containing different concentrations of the I_2_ additive (0, 0.01, 0.05, and 0.1 M) in 2.0 M ZnSO_4_ + 0.1 M ZnI_2_; Figure S2. Photograph of the cell with the carbon cloth and Zn foil as the cathode and anode, respectively; Figure S3. Equivalent electrical circuit used to fit the EIS spectra; Figure S4. Discharge curves of cells with different ZnI_2_ additive concentrations at 10 A·cm^−2^; Figure S5. Photograph of the 2.0 M ZnSO_4_ + 0.1 M ZnI_2_ + I_2_ electrolyte with and without a zinc foil after standing overnight. After standing overnight, the electrolyte in which the zinc foil was inserted turned colorless.
