# Supplementary material for: Insights on Polyidide Shuttling of Zn-I2 Batteries by I3−/I− Electrolytes Based on the Dual-Ion Battery System
Source: Nanomaterials (Basel). 2025 May 14;15(10):738. doi: 10.3390/nano15100738 (PMC12114353; doi:10.3390/nano15100738)
Supplement: Supplementary file 1 [file nanomaterials-15-00738-s001.zip › nanomaterials-3633481-supplementary.pdf]

SUPPORTING INFORMATION

Insights on polyidide shuttling of Zn-I_2 batteries by I_3^-/I^- electrolytes based on dual-ion battery system

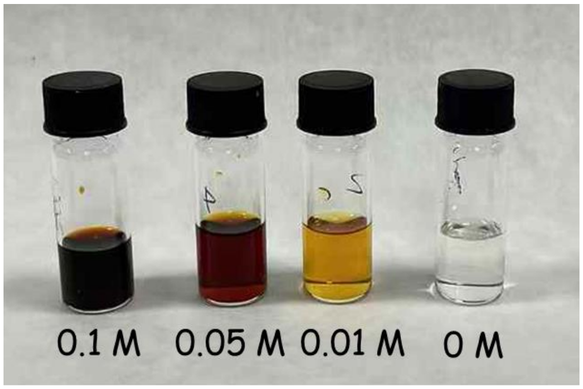

Figure S1. Photograph of vials containing different concentrations of the I_2 additive (0, 0.01, 0.05, and 0.1 M) in 2.0 M $ZnSO_4$ + 0.1 M ZnI_2.

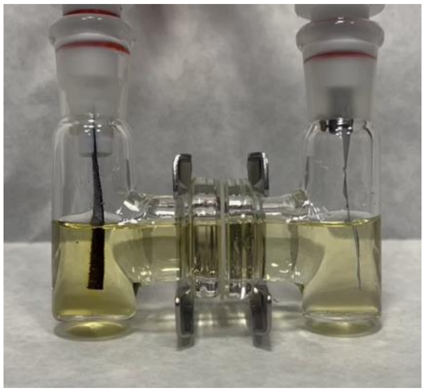

Figure S2. Photograph of the cell with the carbon cloth and Zn foil as the cathode and anode, respectively.

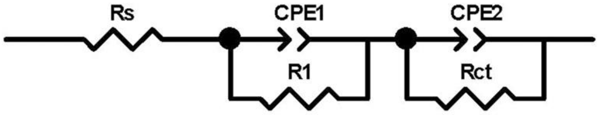

Figure S3. Equivalent electrical circuit used to fit the EIS spectra.

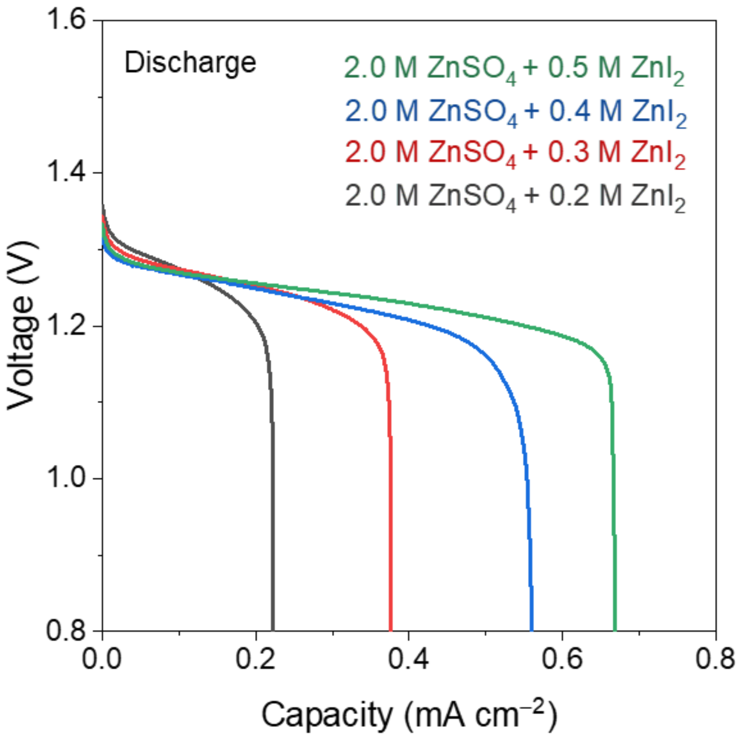

Figure S4. Discharge curves of cells with different ZnI_2 additive concentrations at 10 A·cm^{-2}.

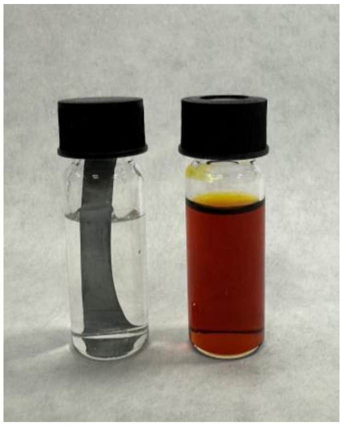

Figure S5. Photograph of the 2.0 M $ZnSO_4$ + 0.1 M ZnI_2 + I_2 electrolyte with and without a zinc foil after standing overnight. After standing overnight, the electrolyte in which the zinc foil was inserted turned colorless.
